# Microglia have a grip on brain microvasculature

**DOI:** 10.1038/s41467-021-25595-3

**Published:** 2021-09-06

**Authors:** Kassandra Kisler, Angeliki Maria Nikolakopoulou, Berislav V. Zlokovic

**Affiliations:** Zilkha Neurogenetic Institute, Department of Physiology and Neuroscience, Keck School of Medicine of the University of Southern California, Los Angeles, CA USA

**Keywords:** Blood-brain barrier, Microglia, Neuro-vascular interactions

## Abstract

Microglia are brain resident immune cells with multiple functions. However, little is known about microglia-vascular interactions. In a recent paper published in Nature Communications, Bisht et al. identify a signalling mechanism that attracts and maintains microglia at the capillary wall. Moreover, they show that microglia regulate capillary vascular tone, playing a more significant role in blood flow regulation than previously thought.

Microglia are the resident immune sentinels of the brain, responsible for the brain immune response as well as a variety of physiological functions including synapse pruning, neuronal regulation, and clearance of cellular and toxic debris^1–4^. However, much less is known about microglia-vascular interactions, particularly under healthy steady-state conditions.

Recent studies using live 2-photon imaging, confocal, expansion, super-resolution, and electron microscopy, suggested that microglia associate with capillary vasculature at all ages and during central nervous system development, notably in areas lacking astrocyte endfoot coverage. They also suggested that motility of juxtavascular microglia declined as astrocyte endfeet more fully ensheathed the vasculature^2^. Others have suggested that during developmental colonization of the retina, microglia migrate to contact the deep nuclear retinal layer of high stiffness, which coincides with microglia bipolarization, reduction in transforming growth factor-β1 signaling and termination of vascular growth^3^. Yet in the adult brain, microglia are an often overlooked component of the neurovascular unit (NVU) —a basic functional unit of the brain coordinating neuronal functions with vascular functions.

The NVU is composed of a collection of functionally interacting cells that form, maintain, and regulate brain vasculature including blood-brain barrier (BBB) integrity and cerebral blood flow (CBF) regulation. This includes endothelial cells, mural cells (pericyte, smooth muscle), glia (microglia, astrocytes, oligodendrocytes), and neurons^5^. Among these cell types, endothelial cells make up a continuous tightly junctioned inner monolayer of the vessel walls forming the BBB, which is surrounded by a basement membrane and other cells of the NVU, as illustrated here at the level of brain capillary (Fig. 1). At the capillary level, pericytes play an essential role in maintaining BBB integrity^5–7^. Interestingly, recent work suggested that microglia could also play a role in repairing damaged BBB^1,8^. One study suggested that microglia migrate rapidly to the site of laser-induced focal capillary wall lesions to seal and repair the damaged BBB, which required G-protein coupled purinergic receptor P2RY12 on microglia^8^. Similar microglia migration to the BBB was observed in the Murphy Roths Large/lymphoproliferation (MRL/lpr) mouse model of systemic lupus erythematosus, which also exhibits BBB breakdown^1^.

**Fig. 1:**
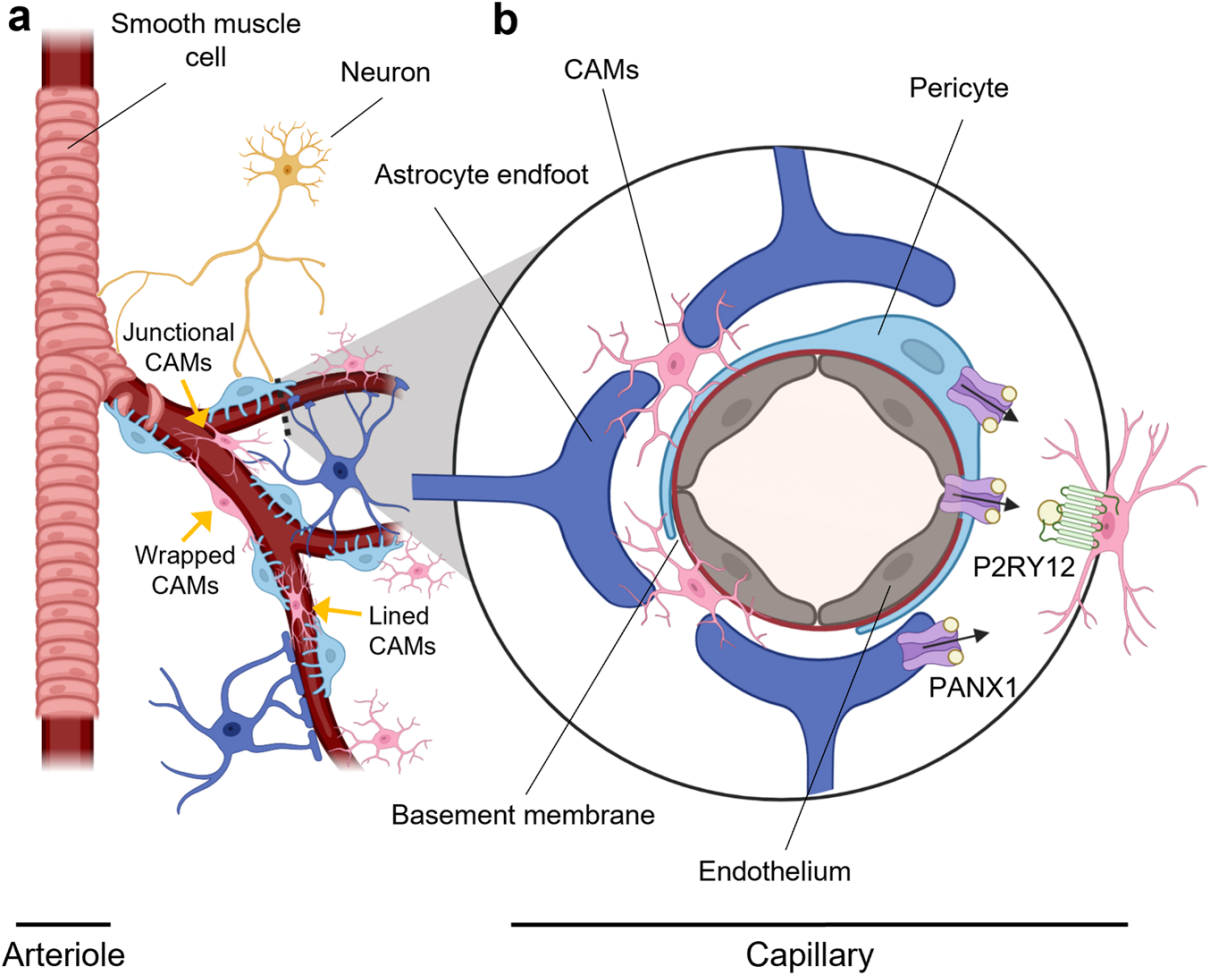
Microglia positioning in the neurovascular unit. **a** Schematic of the neurovascular unit, which is comprised of neurons, vascular cells (endothelial cells, pericytes, smooth muscle cells) and glia (astrocytes, microglia). Endothelial cells (gray) are separated from pericytes (light blue) by the basement membrane (red). Astrocytes make contacts with both pericytes and endothelial cells at the capillary wall with their astrocytic endfeet (dark blue). Neurons (gold) innervate contractile cells of the vessel walls—smooth muscle cells in arterioles, and pericytes in brain capillaries. Capillary-associated microglia (CAMs; pink) make close contacts with brain capillary wall especially in areas not covered by astrocyte endfeet. Bisht et al.^9^ identified three types of CAMs with lined, wrapped or junctional morphologies in relation to brain capillaries. **b** Inset shows brain capillary with P2RY12 (green)-positive microglia that are activated by purines (yellow circles) released from PANX1 channels (purple) from the brain capillary wall likely expressed in endothelial cells, and/or pericytes or astrocyte endfeet. Bisht et al.^9^ have identified a signaling mechanism that attracts and maintains CAMs at the capillary wall.

While these studies shed light on microglia interactions with vasculature in the presence of damage or disease, how microglia interact with healthy adult brain vasculature and the other cells forming the NVU remains elusive. Bisht et al examine the interactions of juxtavascular capillary-associated microglia (CAMs) with brain capillaries and their role in cerebral blood flow (CBF) regulation in vivo^9^. Using 2-photon in vivo imaging of adult mice expressing GFP in brain-resident CX3CR1-positive microglia, the authors first identify microglia cell bodies in close apposition with microvessels, consistent with other studies that observed CAMs^1,2^. Bisht et al also make an effort to systemically classify CAM states based on morphology and somata position relative to capillaries into “lined” CAMs with cell somas laying parallel to the capillaries, “junctional” CAMs with cell bodies at capillary branches, and CAMs that “wrapped” around capillaries with bipolar-like processes. Surprisingly, about 30% of brain resident microglia observed in adult mice are CAMs, resulting in a capillary-associated soma density that is higher than that of astrocytic cell bodies, but lower than pericyte cell body density. Electron microscopy further reveals that the myeloid cell bodies are closely associated with brain capillaries, localized outside the basement membrane that surrounds capillary endothelium, without any interposing astrocytic processes or endfeet consistent with a previous study^2^.

Longitudinal in vivo imaging revealed that most CAMs maintained their location over time, but a few exhibited motility, crawling along capillary vasculature or moving to engage with or detach from vessels. Upregulation of neuronal activity produced by kainic acid (KA)-induced seizure activity did not alter microglia or CAM densities in vivo in cortex, or in hippocampus 48 h after KA administration. Furthermore, depletion of brain resident microglia using CSF1R inhibitor PLX3397 followed by repopulation of microglia after withdrawal of the drug resulted in repopulation of a similar percentage of CAMs along the microvasculature^9^, suggesting that biochemical cues may attract CAMs to the vasculature.

To further evaluate the interaction between CAMs and microvessels, the authors next look at mice with genetic knockout of the microglia-specific purinergic receptor P2RY12^9–11^, which plays a role in microglia motility^8,11^. In *P2ry12*^*−/−*^ mice, the authors observe a reduction in CAM density and slight increase brain parenchymal microglia density, without changes to the percentage of stationary to motile CAMs. To confirm whether the reduced CAM interactions are due to changes in purinergic signaling, the authors next look at the purine channel pannexin 1 (PANX1), which may serve as a source of purines for P2RY12 receptors and is expressed in multiple NVU cell types including endothelial cells, pericytes and astrocytes^10^. The authors observe similar reductions in CAM-vascular interactions and increased parenchymal microglia densities in the *Panx1*^*−/−*^ animals^9^, indicating that the tight interactions between CAMs and capillaries are regulated, at least in part, through purinergic signaling.

Interestingly, these microglia manipulations do not appear to disrupt BBB integrity or alter pericyte or astrocyte coverage. However, the loss of CAMs has a remarkable effect on microvascular tone and CBF. Upon microglia depletion with PLX3397, Bisht et al.^9^ observe a 15% increase in capillary diameter compared to control. This change in vascular tone is accompanied by a 20% increase in basal CBF measured with laser speckle imaging. Furthermore, PLX3397-treated animals exhibit an impaired vasodilatory response to hypercapnic CO_2_ inhalation challenge. *P2ry12*^*−/−*^ and *Panx1*^*−/−*^ mice show similar increases in basal CBF, and impaired responses to CO_2_ inhalation challenge. These observations bring to light a previously undescribed role for microglial regulation of capillary vascular tone.

Currently pericytes are thought to regulate vessel diameter at the capillary level, and by extension blood flow^5,7,12–16^. Pericytes actively participate in neurovascular coupling. Indeed, pericytes have contractile properties that help to regulate basal capillary tone^7^^,^^5^, resulting in rapid increase in regional CBF in response to neuronal activity. Astrocyte-mediated signaling may regulate in part pericyte-mediated CBF changes^14,15^. CAMs preferentially localize along the vasculature in locations where astrocyte endfoot coverage is absent^2,9^, raising the question whether microglia could play a similar regulatory role (Fig. 1). Bisht et al.^9^ show that the changes in hyperemia due to loss of CAMs occur in response to inhalation of CO_2_, a molecule that has been described to act directly on the vasculature, independently of neuronal activity. Therefore, whether depletion of CAMs can alter neurovascular coupling remains to be further investigated. Similarly, any direct microglia signaling to arteriolar smooth muscle cells, another cell type involved in the regulation of CBF and neurovascular coupling (Fig. 1), or signal transduction through endothelial-mediated retrograde signaling from the capillaries^5^, remains open.

Importantly, in the study published in Nature Communications, Bisht et al.^9^ identify a novel signaling mechanism that attracts and maintains CAMs at the capillary wall, regulating the vessel tone. These observations open a host of unanswered questions on the potential roles of microglia in health and disease. For instance, do microglia activated by vascular purinergic signaling exert their influence over CBF directly by detaching from the capillary wall and/or by signaling other cell types in the NVU, such as pericytes or endothelial cells? Could microglia vascular modulation change with astrocyte activation? Microglia and astrocyte disfunction play important roles in neurodegenerative diseases such as Alzheimer’s disease^4,5^. Could aging, disease, or injury change microglia, affecting vascular function? Bisht et al.^9^ show that microglia have a firm grip on brain microvasculature, opening exciting questions to be addressed by future studies.
